# Patient and clinician perspectives on a patient‐facing dashboard that visualizes patient reported outcomes in rheumatoid arthritis

**DOI:** 10.1111/hex.13057

**Published:** 2020-04-09

**Authors:** Lucy H. Liu, Sarah B. Garrett, Jing Li, Dana Ragouzeos, Beth Berrean, Daniel Dohan, Patricia P. Katz, Jennifer L. Barton, Jinoos Yazdany, Gabriela Schmajuk

**Affiliations:** ^1^ Division of Rheumatology University of California ‐ San Francisco San Francisco California; ^2^ San Francisco VA Medical Center San Francisco California; ^3^ Philip Lee Institute for Health Policy Studies UCSF San Francisco California; ^4^ School of Medicine Technology Unit (SOMTech) University of California ‐ San Francisco San Francisco California; ^5^ VA Portland Health Care System Oregon Health Sciences University Portland Oregon

**Keywords:** dashboard, patient‐reported outcomes, rheumatoid arthritis

## Abstract

**Background:**

Poor patient‐clinician communication around patient‐reported outcomes (PROs) is a barrier to the effective management of rheumatoid arthritis (RA). We aimed to develop an RA ‘dashboard’ that could facilitate conversations about PROs and that would be acceptable to a wide range of patients, including English and Spanish speakers and patients with adequate or limited health literacy.

**Methods:**

A diverse group of RA patients along with clinicians from two academic rheumatology clinics joined separate focus groups. We solicited feedback and made iterative changes to mock‐ups of an RA dashboard that visualized PROs using a human‐centred design process. We used the thematic analysis method to identify and characterize themes from the focus groups and used these insights to refine the dashboard.

**Results:**

We conducted six focus groups involving 25 RA patients and three groups with 11 clinicians. Patients and clinicians agreed that the dashboard could enhance communication about PROs and RA disease activity and could promote patient self‐management. Patients varied in their (a) comprehension, (b) preferences for the display and features of the dashboard, and (c) desired uses for the dashboard. Clinicians expressed significant concerns about the logistics of using the dashboard in clinical practice.

**Conclusion:**

Using principles of human‐centred design, we created an RA dashboard that was well‐accepted among patients and clinicians. The ability to customize the data display is important for tailoring the dashboard to patients with diverse needs and preferences. Special attention should be given to feasibility concerns voiced by clinicians.

## 1 INTRODUCTION

The effective use of patient‐reported outcomes (PROs) data is anticipated to play a critical role in improving health‐care delivery, patient experiences with care, and outcomes. In rheumatoid arthritis (RA), a complex chronic condition characterized by joint pain and inflammation, validated PROs have been used over the past several decades to assess levels of RA disease activity and functional status. Routine assessment of PROs is now recommended by American College of Rheumatology (ACR) guidelines, and quality measures to encourage the regular collection of RA PROs have been endorsed by the National Quality Forum.^1^ Treatment algorithms for RA rely on PROs to guide clinical decisions around use of disease‐modifying drugs (DMARDs).^2^ RA is thus an important example of how an application to visualize PROs could be used during face‐to‐face visits to influence care, providing key data for clinical decision making, quality measurement, practice improvement, and research.

In recent years, scientists and clinicians have become more interested in developing patient‐facing dashboards for many chronic conditions, some of which have shown great promise in empowering patients and facilitating self‐management. In diabetes, for example, some mobile applications have demonstrated clinical improvement in glucose control.^3^ PRO‐specific dashboards have also been developed to track outcomes relating to prostate cancer, multiple sclerosis, various surgical procedures, among others.^4^, ^5^, ^6^, ^7^, ^8^ In their pilot study, Hartzler et al found the use of a PRO dashboard created meaningful discussions on quality‐of‐life issues between prostate cancer patients and their clinicians during follow‐up visits. Six RA‐specific dashboards have been developed previously, such as Rheum‐PACER from Geisinger Health System, the Swedish Rheumatology Quality Register, and Rheum4U from the University of Calgary.^3^, ^9^, ^10^, ^11^ These platforms aggregated disease activity measures and PROs, but were designed to support clinician decision‐making, rather than focus on enhancing a patient's understanding of their own disease.^12^

Building on preliminary work by our group to design a first prototype,^13^ the objective of this study was to develop an RA ‘dashboard’ that could facilitate conversations about PROs between patients and clinicians. One key requirement was that the dashboard be acceptable to a wide range of patients, including English and Spanish speakers and patients with adequate or limited health literacy. To this end, we performed focus groups with a diverse group of patients and clinicians, eliciting information to help us tailor this digital tool to address the needs and preferences of patients as well as the health‐care team.

## 2 MATERIAL AND METHODS

### 2.1 Dashboard prototype development

We tested various dashboard prototypes during patient and clinician focus groups to arrive at an optimal dashboard design. We used the principles of human‐centred design, which aims to integrate the needs of people, the possibilities of technology, and the requirements for success, and is used widely in the development of health‐IT tools.^14^, ^15^ We utilized a modified version of A/B testing (ie, comparing two versions of the dashboard with only one major difference side by side) with patients where they provided verbal feedback on prototypes ranging from rough sketches on paper to interactive mock‐ups on a tablet computer.

**FIGURE 1:**
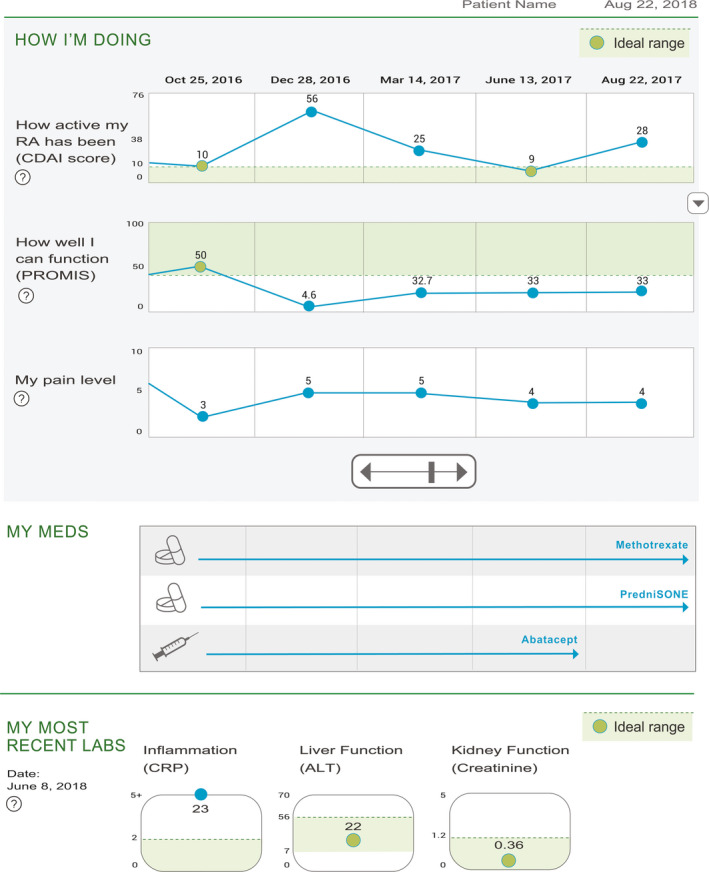
Prototype A mock‐up of RA dashboard. Sections include ‘My RA symptoms’, representing the CDAI disease activity score, ‘How well can I function’ showing PROMIS physical function scores, and ‘My pain’. Medication and laboratory information are also displayed

The first prototype was previously developed by our investigative team using principles of human‐centred design.^13^ In brief, we conducted patient and clinician semi‐structured interviews, small focus groups of English speakers, and heuristic evaluations in rheumatology clinics to generate the first prototype (Figure 1, Prototype A). The first prototype included visualizations of three PROs: (a) a measure of RA disease activity; we chose to display a validated instrument to measure RA disease activity that is used routinely in our clinic, the Clinical Disease Activity Index (CDAI). The CDAI is a composite score that includes a tender joint count, swollen joint count, patient global assessment score, and physician global assessment score that has been endorsed by the American College of Rheumatology,^16^ (b) a measure of functional status; we chose to display a validated instrument to measure functional status that is used routinely in our clinic, the Patient‐Reported Outcomes Measurement Information System (PROMIS)‐physical function scale. The PROMIS‐PF is a patient‐reported measure that includes questions about activities of daily living and level of physical activity and has been endorsed by the American College of Rheumatology.^17^), and (c) a pain score that is collected during routine clinical care on all patients. These were each displayed in series on a graph with level or score for the PRO on the y‐axis and time on the x‐axis. The five most‐current time points were visible to begin with, and scroll buttons allow users to see historical scores. Relevant medications used specifically for treating RA were also shown as solid bars (representing time spent receiving the drug) underneath the PRO measures. Key laboratory results, including a measure of inflammation (CRP), liver function (ALT), and kidney function (Creatinine) in relation to normal ranges, were also displayed. Prototype A was translated into Spanish by a native speaker on the research team (GS).

**FIGURE 2:**
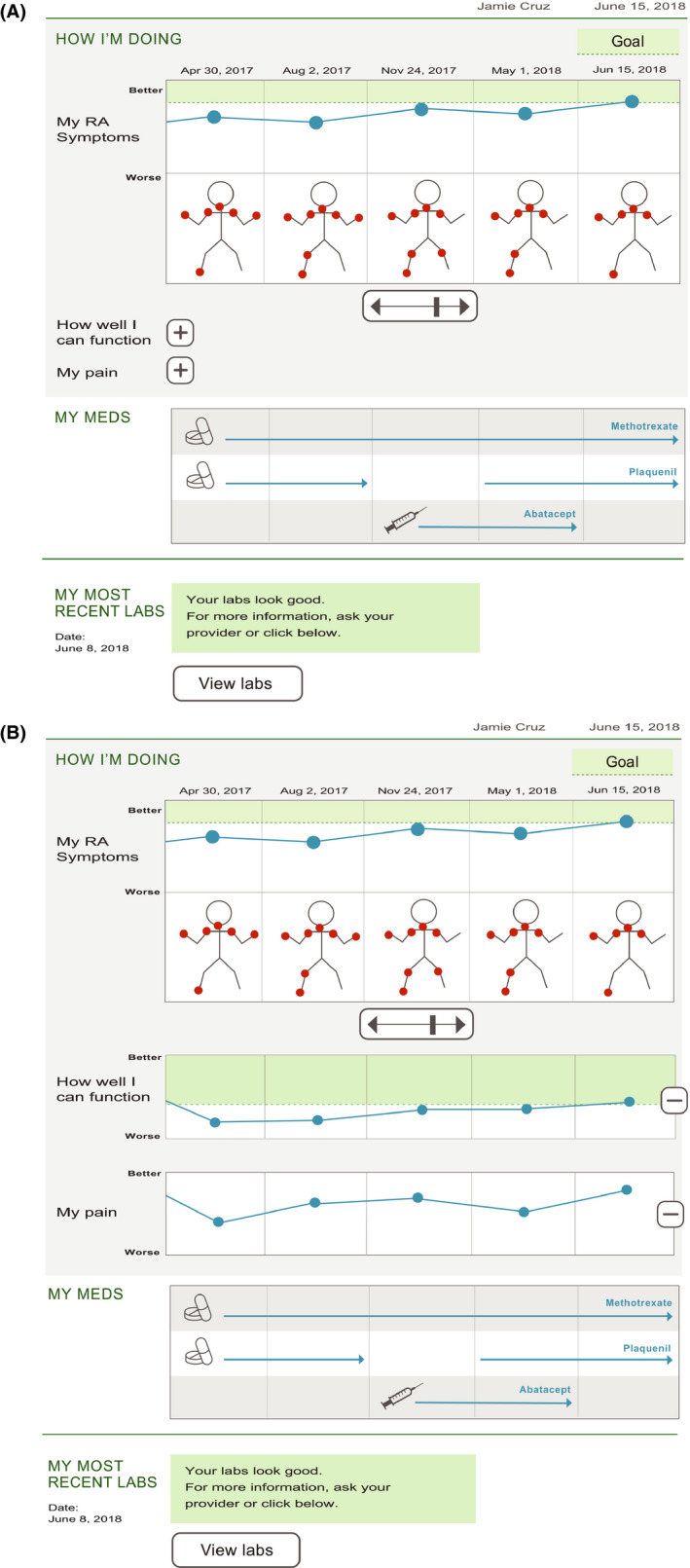
Prototype B mock‐up of RA dashboard, showing collapsed (A) and expanded (B) views. Sections include ‘My RA symptoms’, representing the CDAI disease activity score along with homunculus with red circles representing areas of disease activity, ‘How well can I function’ showing PROMIS physical function scores, and ‘My pain’. Medication and laboratory information are also included, but collapsible in case less detail is desired. Key changes from earlier prototype include flipping the direction of ‘good’ for the disease activity score; removing reference to ‘CDAI’ and ‘PROMIS’; the addition of the homunculus; making all sections collapsible; and changing ‘ideal’ to ‘goal’ and ‘better’

The second set of prototypes (Prototype B, see Figure 2A,B) were developed in response to the early patient focus groups (groups 1, 2, 3), and refined and tested for acceptability in the later focus groups (groups 4, 5, 6). In general, the second prototypes were partly customizable by making each section of the dashboard expandible/collapsible so that each PRO (or laboratory) could be displayed one at a time, based on a patient's preferences. Additional key changes from the first prototype included (a) adding a homunculus (a cartoon humanoid creature with red dots in the extremities representing areas of joint inflammation) to correspond to swollen joints counts, with historical data available by scrolling to the left; (b) changing the orientation of the disease activity visualization so that ‘better’ was in the ‘up’ direction; (c) removing references to ‘ideal ranges’; and (d) adding ‘pop‐up’ explanatory text to provide more details on a PRO or test result.

### 2.2 Focus group participants

Human‐centred design prioritizes the iterative and in‐depth engagement with potential users of the product under design in order to ensure that the product is sensitive to the user population's hopes, needs, capacities and preferences.^18^ Focus groups are an effective and commonly used method that allows designers to obtain this information. Guided by principles of human‐centred design, we conducted a series of six focus groups with patients and three focus groups with clinicians. Patients were eligible for the study if they had a diagnosis of RA, were ≥18 years old, were primarily English‐ or Spanish‐speaking and received care in rheumatology clinics at either a university hospital (tertiary academic referral centre) or in a county hospital based in California. Patient focus group participants were recruited via purposeful sampling by telephoning RA patients from these different clinics with previously documented adequate or limited health literacy. Modest compensation was provided for patient participation. For the clinician focus groups, eligible clinicians included fellow trainees, faculty rheumatologists and other members of the health‐care team (eg, nurse practitioners or medical assistants) who practiced at these sites.

Patient characteristics, such as their age, sex, race/ethnicity and language preferences, were obtained from the electronic health record (EHR). Patient's health literacy was assessed based on the Short Test of Functional Health Literacy in Adults (S‐TOFHLA), a validated measure of health literacy.^19^ Patients scored ‘marginal’ or ‘inadequate’ were deemed to have limited health literacy.

### 2.3 Focus groups

Patient focus groups were formed based on patient language preference (English and Spanish) and health literacy level. We conducted separate groups based on health literacy because we hypothesized that adequate vs limited health literacy patients might have distinct preferences for dashboard content and presentation. Clinician focus groups included rheumatology clinical trainees and faculty rheumatologists and a nurse practitioner. Focus groups lasted 60‐90 minutes and were held in a conference room near each of the rheumatology clinics. Focus groups were facilitated by native speakers in each language who had qualitative research and facilitation expertise (SBG, CK). The primary facilitator, designer and research assistant attended all focus groups to ensure consistency of focus group conduct and note‐taking across groups (SBG, DR and JL).

At the beginning of each focus group, participants were assured that their responses would be aggregated for presentation and that standard steps would be taken to ensure confidentiality of the data. Verbal consent was obtained. Facilitators encouraged all individuals to participate; invited participants to voice diverse experiences and ideas rather than seek to find consensus in the group; and explicitly solicited participants’ concerns about the dashboard and its use in clinic settings. The study team provided paper‐ as well as iPad‐ and laptop‐based prototypes. Participants were encouraged to write and draw on the paper versions. All focus groups were audio recorded. Research staff collected annotated copies of the prototype at the end of each focus group. After each focus group, the study team shared notes and discussed emergent themes.

All focus group guides were developed in English by an expert in qualitative research (SBG) and authors who had participated in qualitative research activities with RA patients during an earlier phase of the study^13^: though patients were not directly involved in guide development, the patient guides were informed by preliminary data the team had collected from patients in prior semi‐structured interviews and focus groups. Native speakers on the research team then translated (GS) and back‐translated (CK) the guide for the Spanish focus groups. Consistent with human‐centred design principles, focus group guides were designed to encourage patients to think concretely about their individual experiences (past, present and future) when evaluating the dashboard rather than discussing the dashboards abstractly or for a ‘typical user’. Patients first discussed their experiences in the RA clinic, focusing on their expectations and needs during appointments. The RA dashboard was then introduced, and patients described their understanding of the dashboard and shared feedback on whether the dashboard adequately represented factors important to them and their disease. Patients were asked to discuss how the dashboard might be used for managing their disease, and how it might change their future appointments or self‐care. They were also given an opportunity to share thoughts on different formats of the dashboard.

Cognizant that some participants might feel pressure to agree with group sentiment or to embrace the dashboard prototype in front of its designers, we sought to capture patients’ private views as well. We developed an anonymous, 2‐item survey administered at the end of the focus groups, that asked patients to indicate whether they would like to use a dashboard themselves at their next visit and what dashboard format they would prefer (eg, in‐clinic computer screen; home‐based computer screen; mobile device; paper). The survey required less than a minute to complete. Results were descriptively tabulated and summarized in the text below.

The focus group guide for clinicians was similar in format to the patient guide and focused on how the dashboard could enhance communication with patients. Guide designers solicited the feedback of clinicians on the research team in order to assess and improve the relevance, clarity and appropriateness of the guide questions. Clinician focus groups began by discussing clinician goals and challenges for RA face‐to‐face visits, followed by the introduction of the dashboards. Clinicians gave feedback on the dashboard, commented on potential application in their practice, and suggested improvements.

### 2.4 Focus group analysis

All audio recordings were professionally transcribed and, for the Spanish focus groups, translated. An author with expertise in qualitative methods (SBG) conducted iterative inductive and deductive thematic analysis^20^, ^21^ during and after data collection to identify themes relevant to patient or clinician perspectives on the dashboard and its use. Deductively, for example, we investigated expressions of interest in and understanding of the dashboards in each group, as these were phenomena the team had a priori sought to study. Inductively, we explored what factors participants cited when describing why they would or would not be interested in using the dashboard. We used analytic memo‐ing to document preliminary themes within and across focus groups; to explore relationships between themes (eg, interest and understanding); and to integrate the interpretations of other team members who observed and took notes on the focus groups. Other members of the team (LHL, JL, GS) reviewed the data and preliminary analyses and collectively evaluated, refined and affirmed the proposed themes. Members of the team who attended the focus groups (SBG, JL, DR, CK) reviewed the data and preliminary analyses and collectively refined and affirmed these themes.^20^, ^21^ This study was approved by our institution's Institutional Review Board. The data that support the findings of this study are available from the corresponding author upon reasonable request.

## 3 RESULTS

### 3.1 Patient focus groups

Six‐patient focus groups were conducted, four in English and two in Spanish, involving a total of 25 RA patients. Characteristics for the patients are shown in Table 1. Eighty percentage of patients were female; average age was 59.9. There were 15 English‐speaking and 10 Spanish‐speaking patients. Sixteen patients had adequate health literacy and nine patients had limited health literacy (three groups were majority adequate health literacy; three groups were majority limited health literacy). For nearly all groups, patient attendance represented approximately 50% of the patients recruited for the event.

**TABLE 1:** Characteristics of focus group patient participants

FG #	ID	Age range (y)	Gender	Race/ethnicity	Health literacy	Primary language	Clinic site
1	P1	60‐70	F	Hispanic	Limited	English	County Hospital
1	P2	60‐70	M	Black	Limited	English	County Hospital
1	P3	60‐70	F	Hispanic	Limited	English	Academic Center
2	P4	60‐70	F	Black	Adequate	English	Academic Center
2	P5	70‐80	F	White	Adequate	English	Academic Center
2	P6	60‐70	F	Hispanic	Adequate	English	County Hospital
2	P7	60‐70	M	Black	Adequate	English	Academic Center
3	P8	60‐70	F	American Indian or Alaska Native	Limited	English	Academic Center
3	P9	60‐70	F	Black	Limited	English	County Hospital
4	P10	40‐50	M	White	Adequate	English	Academic Center
4	P11	70‐80	M	White	Adequate	English	Academic Center
4	P12	30‐40	F	White	Adequate	English	Academic Center
4	P13	60‐70	F	Hispanic	Adequate	English	Academic Center
4	P14	60‐70	F	Asian	Adequate	English	Academic Center
4	P15	60‐70	M	White	Adequate	English	Academic Center
5	P16	60‐70	F	American Indian or Alaska Native	Limited	Spanish	County Hospital
5	P17	40‐50	F	Hispanic	Limited	Spanish	County Hospital
6	P18	40‐50	F	Hispanic	Limited	Spanish	County Hospital
6	P19	60‐70	F	Hispanic	Limited	Spanish	County Hospital
6	P20	60‐70	F	Hispanic	Adequate	Spanish	County Hospital
6	P21	60‐70	F	Hispanic	Adequate	Spanish	County Hospital
6	P22	50‐60	F	Hispanic	Adequate	Spanish	County Hospital
6	P23	40‐50	F	Hispanic	Adequate	Spanish	County Hospital
6	P24	40‐50	F	Hispanic	Adequate	Spanish	County Hospital
6	P25	60‐70	F	Hispanic	Adequate	Spanish	County Hospital

We identified four main themes across focus groups: (a) acceptability and interest in the dashboard, (b) comprehension of the data displayed in the dashboard, (c) preferences for dashboard display and features, and (d) suggestions for potential uses. Illustrative quotes are presented in Table 2.

**TABLE 2:** Quotes and thematic findings from adequate health literacy and limited health literacy patient focus groups conducted in English or Spanish

Themes	Adequate health literacy group quotes	Limited health literacy group quotes
A. High levels of acceptability and interest among patients	‘I was just diagnosed with this disease, but it would be really helpful to see my progress by receiving this kind of information’. (P23, FG6) ‘I think it would be positive because it shows you your progress, what works and what doesn't work in the long run’ (P7, FG2) ‘This is useful because a lot of times I go in and I don't remember how I was feeling six months ago… or a year ago, and how it is comparable to that. So it's kind of nice to see the tracking here’. (P11, FG4)	‘I'm for it…I would go with it just like it is…I'd like to see that every time I go see my doctor… I can be prepared‐ how to take care of my disease and how the doctor could better see how I'm doing with that information’. (P2, FG1) ‘Well, it'll show me that I'm doing what I'm supposed to be doing ‐ taking my meds, exercising, doing the right things. You know? And I can see that I'm doing the right things by this chart here’. (P2, FG1)
B. Variation in the comprehensibility/accessibility of the dashboard; improvements in understanding followed explanation	‘It took me a long time to understand it… I think, ‘What are all these numbers doing here?’ (P5, FG2) ‘I have no idea. I have no idea of what this really is’. (P22, FG6) ‘I just glaze over looking at numbers. It took me a very long time to figure out how to deal with this because I'm not a numbers person’ (P5, FG2) ‘You've got to come up with a tutorial. When people go visit their rheumatologist appointment, ask them do they have five minutes to watch something and to learn. That's how it's going to get over. But you can't just put it out like this’. (P4, FG2) ‘It feels like a really dumbed‐down child version, like this is the version for the children that adults are taking care of’. (P10, FG4) ‘At the beginning it was a little confusing, but later we were talking and it cleared up our confusion, because now I understand this chart’. (P6, FG2)	‘The truth is I don't understand that one well because they didn't draw the [homunculus]… If the five fingers are hurting, they draw circles’ (P17, FG5) ‘This really isn't pleasing to my eye to look at, really… But I don't know what to expect when I'm looking at something like this about me. Because I do fill out these forms every time I go to the doctor. I do have blood work two or three times a year… And they do have a thing about my meds.’ (P8, FG3) ‘It's not unappealing. It's very childish, actually’. (P9, FG3)
C. Diverse preferences on dashboard display design and features	‘They should scale the circles [on the homunculus] based on how bad it is’ (P12, FG4) ‘It would be better to have numbers, because we can measure the amount’. (P20, FG6) ‘I think it's confusing that good [CDAI] is on the bottom and the [bad] CDAI score on the top….The axis [should be] scaled better. I get the [PROMIS score] because that's a percentage, but that should be stated’. (P12, FG4) ‘The ideal range for me isn't nowhere near the ideal range for the general population. So, I think that it needs to be personalized because a person that has as much as I do don't ever get normal…Everything is not a general thing’ (P4, FG2) ‘“My meds” is a waste of space. We all know what medications we're taking. We could put other more useful information, like labs over time’. (P12, FG4) ‘I want all of the information. I want access to all of my labs over time. I want access to my doctor's notes, which I currently have no way to get access to in any way. I want access to the notes that they send each other, which I also have no way of getting access to over time. I mean, this is like comics, right? For somebody who's an active enough patient managing care, like who is active enough in managing their own care, this is almost, yeah, like a caricature’ (P12, FG4) ‘The best thing would be if we could customize it….I want to see CRP, sed rate… because that's what I care about. (P12, FG4) ‘You can correlate the meds your taking during the times you're having trouble and no trouble, which I guess would be significant’. (P15, FG4) ‘So if they had all of these things plus all the other things we mentioned like fatigue and breaking out these components and stress…then we could line up the blood levels on the same axis and then show instead of them like this maybe we did like an arrow, like here's where you started prednisone, and here's where you got your cortisone shot in your knee. And then we could ‐ and obviously you can't display all those things at once so you could let us pick what we're going to [see] at any moment. (P12, FG4)	‘I would draw it with a person there and I would draw marks for the pain and its intensity, bigger or smaller, with different colors to differentiate it. Inflammation is red and pain is blue, for example…’ (P16, FG5) ‘The meds ‐ what are they there for? We know what kind of meds we take. We don't need [this]’. (P9, FG3) ‘Meds are important. Especially if [you're like] me, I go back five year meds. Okay? Because what did I take three years ago that I did, that I didn't ‐ that I took five years ago, and it didn't work. I want to know that time lapse of what was then and what I can't take now’. (P8, FG3) ‘I like to know what my kidney is and what my liver is. That's important if you have RA. And for the CRP, I can feel it’. (P9, FG3) ‘The labs don't mean nothing to me… [My doctor] tells me the main things, and that's it. I don't care after that point’. (P8, FG2) ‘My pain level ‐ it's like neutral… Because… mine's never been so high or so low that it hasn't been managed. So I wouldn't even count that one. But that's me’. (P8, FG2) ‘What I would like to know… is [what] the doctor's comments are written down (P9, FG3) ‘It would be very nice to see doctor's notes and your allergies’. (P9, FG3)
D. Interest in enhancing the dashboard to support more resources/capabilities	‘[It would be helpful] if it shows when things are not in your range and it's starting to be where it's dangerous or you should see a doctor, that it pop up on your screen or you get a telephone call or something’ (P4, FG2) ‘I have a problem with my health that is not addressed in any of this, but I don't know that it could be… The symptoms that I have with RA is flares that take the form of fatigue where I get exhausted and need to take naps… This is not reflected anywhere I think in this … and I would kind of want to track that’ (P5, FG2) ‘For me fatigue is a big one, which they actually do not ask about on their little form, but would be a key variable for me to track how I'm doing… It doesn't always go with joint pain, either’. (P12, FG4) ‘I [want] something that tracks stress levels because… sometimes my stress will go up to mask when I'm flaring or joint pain with everything’. (P10, FG4)	‘I think doctors, nowadays, they don't have enough time when we go there to pay attention about the food… you have this study on your own, I would do that. And I do something. And I discuss by myself. It says, okay, and say, "I may notice it, doctor. And in three months I didn't see you, I've been eating this and this and this and this."’ (P1, FG1) ‘I'm thinking about the journals. And I think about food. Fruits ‐ what kind of fruit, what kind of meat, the time that I eat. And I feel like that is the only one time we will see what is going on’ (P1, FG1) ‘[We would like to] know when the disease becomes more severe and why, if it's because of the food we eat or because we don't work out, if it's because of the concerns, if that also causes more pain and inflammation. We would like to know that: stress, depression, not working out… All those things we all have, how they affect the disease over time’ (P16, FG5)
E. Diverse potential uses	‘I would share it with my son because he's the one who knows. I only have a son and he's the one who always knows about everything. I show him everything…Because he wants to know everything’. (P21, FG6) ‘it's an option for us to be able to receive it and share it with our family, not for us to pretend we're sicker but for them to know what we suffer from’. (P25, FG6) ‘I noticed a lot of times when I go to the doctor, when one of my specialists, my spinal doctors or something, wants to do some type of procedure or whatever, he'll say no because my arthritis is too active. So, this would help a lot. I think it would help you as well as your other physicians as far as to be able to check the activity of your arthritis’. (P4, FG2) ‘This would be useful for me to take the information to discuss with my PCP or other doctors because the computer systems in each hospital sometimes not linked. So my PCP sometime doesn't get the information from my rheumatologist. So I don't know whether that can be done internally but if I have this information… I can at least print out a hard copy… and [bring it to my clinicians] that's not in this hospital’. (P14, FG4) ‘if you're able to access it from home, that you don't have to go to the doctor to find out that your stuff is acting up’. (P4, FG2) ‘In the clinic, it gives you something to discuss with your doctor or give you information that you're not sure, because I used to didn't know, "What does that mean that it's active? I know I've got it, but what do you mean? Is it doing something else?"’ (P4, FG2) ‘I think this system should be online integrated with MyChart’. (P14, FG4)	‘I would be happy because I would check from home if the doctor has anything to write about me and I wouldn't have to ask him. Because sometimes when they do my labs, I have to wait until I have an appointment with my family doctor, which is sometimes three or four months later’. (P17, FG5) ‘If they give it to me in a paper, I can read it before and if there is anything I don't understand, I can underline it and ask about it. Because, as I said, we get very little time for our appointments and we sometimes feel cut off. So if they give me the paper, like they say, everything holds up on paper’. (P17, FG5) ‘By the time you're done with your doctor's visit, this could be printed up on one of the final sheets of your summary sheet… and we can see "Oh, this is what I answered today. Oh, okay. I get it. Now I see I'm getting better or I'm not doing so well.”’ (P8, FG3) ‘Not online, though… I trust nothing online’ (P9, FG3)

#### 3.1.1 High levels of acceptability and interest in dashboard use

Rheumatoid arthritis patients in the focus groups were generally positive about the dashboard and desired to use it for RA management. Patients described it as ‘helpful’, ‘useful’ and ‘interesting’. Some patients saw it as a repackaging of information with which they were familiar; others felt it would give them novel information about themselves and their disease. Spanish‐speaking and limited health literacy participants were more likely to consider this information new or more accessible to them than what they were used to. This support and interest emerged across all patient focus groups (Table 2A).

In the anonymous summary survey, 21 of 24 patients expressed that they would like to see their own data represented in a dashboard at a future appointment. The remaining three patients responded ‘maybe’. Most patients indicated that they would be interested in using a dashboard similar to one or both prototypes. Very few patients indicated that they would not use the dashboard unless it were substantially modified. Many patients, especially adequate health literacy patients but also some limited health literacy patients, expressed preference for the presentation of more detailed information.

#### 3.1.2 Variation in the comprehension of the dashboard

Many patients found the dashboard difficult to understand at first glance. This initial confusion was more common among limited health literacy patients but not exclusive to them. A few patients were able to understand the dashboard nearly immediately; others after a couple minutes of study or talking through the content. Several patients, particularly in the limited health literacy and Spanish‐language groups, did not notice or understand the longitudinal nature of the data from left to right nor the temporal connection between the different graphic elements, particularly the timing of the medications tracking with the timing of the symptoms. At least one limited health literacy patient found the design ‘not pleasing’. A few patients misinterpreted the line drawn between two data points to mean information from between the visits. Some patients did not understand the dashboard at all until focus group leaders or other patients explained the sections. One adequate health literacy patient explained, ‘I’m not a numbers person’. Once patients understood the organization of the dashboard, however, they were able to engage with the information and viewed the dashboard more positively. Others communicated support for the concept of the dashboard, even before they achieved comprehension. Another patient with adequate health literacy suggested developing a clinic‐based video tutorial for the dashboard, which other patients in the focus group supported (Table 2B).

#### 3.1.3 Diverse views on dashboard display and features

Patients were divided in terms of preferred dashboard style, with some patients preferring Prototype A (which was more numerical and displayed a large amount of detailed information) and some preferring Prototype B (which had a simpler design with more graphics; see Table 2C). A few patients (both adequate and limited health literacy) had a strong negative reaction to the simpler prototype, stating that it was, for example, ‘childish’. In general, there was more support for more detailed information and more complex design in the adequate health literacy groups, but this preference was expressed by some limited health literacy participants as well. Many patients expressed the importance of being able to customize the dashboards to their needs. Some patients disliked the objective ‘ideal range’ and ‘goal’ targets because they did not expect to achieve them given the severe or longstanding nature of their disease.

Patients expressed interest in expanding the dashboard capabilities to include tracking other events between appointments, including RA flares, medication side effects, fatigue, sleep, stress, diet, smoking and exercise (Table 2D). Several patients mentioned that they currently keep a journal of all their RA symptoms and that they envisioned using the dashboard in a similar capacity. Other patients—regardless of health literacy level—wished to use the dashboard as a portal for accessing more information about their medications (mainly side effects), disease management (ie, diet and exercise), clinician's interpretations of how they were doing, and clinician notes.

#### 3.1.4 Diverse potential uses

Patients expressed the desire to use the dashboard to further not just their own understanding of their disease process, but also to help with communicating their experience of their disease to other clinicians or family members. Many expressed that the dashboard could help enhance clinician understanding of their condition and improve coordination of care between multiple specialty clinicians and hospital systems. Patients also thought the dashboard could help them communicate about their disease with their family members. One mentioned that the dashboard would help validate her symptoms to her family and friends (Table 2E).

The importance of being able to share the dashboard with others is reflected in the summary survey, where many patients indicated interest in being able to access their dashboards regularly. Most patients desired a paper version of the dashboard that they could take home with them and show their families or other clinicians. Half of the patients wished to have the dashboard accessible on their personal smart phones, but this was less often mentioned by limited health literacy and Spanish‐speaking patients. About half of the patients also wished to access the dashboard online.

Many patients felt the dashboard could enhance their understanding of their symptoms and disease trajectory, which could in turn help them with self‐management. In particular, patients were interested in tracking disease activity with lifestyle modifications such as dietary changes and exercise. Patients also felt that the dashboard could help them better prepare for their appointments and help focus the discussion with clinicians, particularly in the context of rushed visits. This aspect of use was particularly important to Spanish‐speaking patients, who described constrained communication opportunities with clinicians. Many of these patients felt that the dashboard would help minimize the number of questions that they would need to ask their clinicians. Spanish‐speaking patients did not anticipate challenges in using the dashboard with an interpreter.

### 3.2 Clinician focus groups

Eleven clinicians participated in three focus groups. Eight women and three men differed in their level of training and included three faculty rheumatologists, six rheumatology clinical fellows, one nurse practitioner and one medical assistant. Most participating clinicians practiced at the university hospital, although one group included clinicians from the county hospital as well.

#### 3.2.1 High levels of interest in dashboard use

Most clinicians were supportive of the concepts presented in the dashboard (Table 3A). Many believed it would enhance their ability to communicate with patients and to show them their progression or improvement over time. Some expressed how showing both disease activity and pain on the dashboard would help distinguish what could be treated with RA medications vs not. Clinicians also saw the dashboard as a potential method for aggregating data from various sources (Table 3B). Many mentioned that aggregating information about medication history, number and location of swollen joints, historic laboratory data and imaging results could help with continuity of care and hand‐offs between clinicians. Some thought that a ‘snapshot’ of relevant information for a particular patient would make their own medical decisions easier. A few clinicians thought that two different dashboards would be more effective—one for physicians with more detailed information and another for patients that was more limited.

**TABLE 3:** Quotes and thematic findings from clinician focus groups

Themes	
A. General support of the concept of the dashboard, to enhance communication with patients	‘Having spoken to a lot of RA patients throughout the years and given presentations to patients, I think this would be a very awesome tool… because patients always want to know, “Where am I at with my disease?”’ ‘I think it's a great coaching piece for MAs and the patients as well. Because a lot of patients ask us, what are we really doing this PROMIS score. Why do I have to do this every time that I come into clinic?’ ‘A lot of the patients…ask me… “How was my last lab? What was it related or correlated to the previous one before that?” So, pulling that up on [the computer] and showing them the trend line [would be] such a cool tool’. ‘[This dashboard can help] correlate[e] that their pain level is irrespective of their exam. Because there are so many patients who come in and say, my RA is flaring. When the reality is if you can show them, well, you know what, your joints are actually better and you're functioning…you're actually doing well and we've got all the right meds and your labs look great. Let's talk about the pain, and let's see what else we can do because it sounds like it's outside of your RA now’. ‘I think it would be most helpful when we're counseling around continuing medications. The patient that comes in wanting to stop medications, if you had a way visually to say well, actually, I really do think this has been helping you. I know we're not necessarily in a perfect scenario and your pain level isn't zero, but look what we've been able to achieve. I think that's a helpful visual tool’. ‘It's nice because it compiles a few things in the same place’
B. Interest in using the dashboard as a tool primarily for clinicians	‘Often the fellows are having to inherit a patient who's been cared for by many different people. And especially for patients who have a long‐standing disease who are complicated and who have been on a lot of medications, sometimes things were stopped for unclear reasons. … So, having that historical medication information that's accurate with when it stopped, why it was stopped, etc, would…potentially improve safety for patients in treatment’. ‘The homunculus makes a really big difference because it does help us as providers also see, gee, where did they hurt last time and are they better, any worse’. ‘I think these details that the other people are discussing kind of point to the importance of probably having a different dashboard for the physicians as opposed to the patients, because I think for us to be able to use this in a way that would help us in making a treatment decision for example, we do want to see more granular detail. Which would include the trends in the labs, and also the dosing of the medications. Whereas, if you add all of that detail into a dashboard for the patients, it's probably going to be overwhelming; and it might just not be interpretable because they're coming from such a different background’. ‘I like the idea of being able to click through a few labs. Although, I would need to see more than these three labs for my own assessment of their status. And I definitely don't want to be looking at labs in two different places in the same visit’. ‘I think also it would help me in engaging my threshold to change a medication as well. You kind of do this as you're pre‐charting and…looking back at the last three to four visits over the last year… So being able to kind of pull this up automatically [on the dashboard], actually would I think be helpful’.
C. Scepticism that the dashboard would enhance communication beyond their existing discussions with patients	‘I feel like patients retain so little in a visit because it's really overwhelming… I don't know for my practice, how useful [this dashboard] would be. I find it's more useful to sit with them and draw it out together’. ‘I may not use this as much as with someone who has active disease, [when] my agenda would be to increase their methotrexate’. ‘I think what I would… print it out, so the patient has something physical and then I would probably draw in… here we started with three [pills], and then here we have six [pills]. That's usually how I practice, anyway’.
D. Interest in enhancing the dashboard to support more resources/capabilities.	‘Something that I was thinking, too, is can you get the homunculus in here, too? The actual picture with the joints that are shaded? It's sort of going back to the [picture thing], but these are a lot of lines and dots. And for patients with [limited health literacy]… pictures [will be better]’. ‘I do think this might get confusing…the fact that the [CDAI and PROMIS] go in opposite directions. I think for patients, that might get confusing to understand that. Intuitively for me, at least, I would think up is better and down is worse’. ‘I don't really use the PROMIS when I make treatment… [But] the nicest part about [the PROMIS] is that it's a little bit easier for patients to understand rather than CDAI, as a gauge of like how well their RA is being managed’. ‘All of these [elements] depend heavily on the subjective pain sense of the patient, which I don't think is that uncommon that it may be quite different than our RA‐focused specific disease assessment. Having at least one visual that was maybe more based on the physician's assessment would be [helpful]’.
E. Concern about the quality and availability of EHR data to be imported into the dashboard.	‘[Extracting automatically from the medication list] would make me nervous, and maybe that's a good thing that it would really encourage us to be making sure that the medication list is always up‐to‐date. I know that sometimes it's not, and then I would worry about that being confusing for patients’. ‘The ability to edit the medication… comes in especially when you've written somebody for three [tablets] and they've been really taking however many [tablets]… It would be more useful for us to know what they're actually taking, versus what the prescription actually says’. ‘The MAs flag [medications] for the providers to remove. That doesn't get done, so it's still on the list and it's an ongoing issue unfortunately’. ‘Unfortunately, [labs done at outside labs] will never show in the EHR. They're just scanned or printed from like a paper form, and scanned into the EHR. So, that will never be abstracted’.
F. Apprehension about how the dashboard would fit into their work flow.	‘With those patients where it is already always really tight on time, I would kind of need to think about whether or not this is going to be helpful or harmful for time management. Because it could bring up a need to do a lot of explaining, since [a lot of them] probably haven't heard of the word CDAI or PROMIS, and so kind of getting into defining all of the different pieces of the graph and what those measurement tools mean and what's good and what's bad. I don't know if I would do that for everybody’. ‘if a patient is late to their appointment, they don't finish their PROMIS form, and the provider finishes the visit, how will we print out everything and get it to them on time? Maybe it can cause a delay in a rooming situation’. ‘Just pulling up another application and presenting this to the patient for the first time. They'll be like, oh, what is this? So, you might have another like five, ten minute conversation just about this – [or] anything that we're implementing’. ‘In the beginning, it would take a lot of time just to explain it when they're first rolled out, but I think afterwards, it probably won't be. And as you get new patients coming in, we can have the MAs talk about it right as they go in’ ‘I think it's a good tool that patients would love. Having given talks to patients before with RA disease, they never really quite know how well they're doing because they gauge it on their pain level. And so, seeing all these identifiers pulled open for them would be helpful. And I know as a provider, I don't have time to go over everything I just told you about… Here's a new program… let my MA explain it to you. It sounds like it would be a much more viable option to be able to [use the dashboard better]’.

#### 3.2.2 Scepticism about utility of the dashboard

Despite interest in the content and potential uses of the dashboard, a few clinicians expressed significant scepticism that they would use the dashboard during face‐to‐face visits with their own patients (Table 3C). Several clinicians thought that the dashboard would be overwhelming to patients and that they would prefer to continue using their current strategies of writing down medication plans during the visit. Even among enthusiastic clinicians, some would want to use the dashboard only in limited cases, for example, when recommending a medication change.

#### 3.2.3 Interest in enhancing the dashboard to support more capabilities

Similar to the patients, clinicians were also very interested in customizing the dashboard to their own needs and recommended that it be designed to present more detailed information (Table 3D). Many clinicians wished for additional or enhanced resources in the dashboard, including patient‐facing educational materials about medications, or other tools to assist patients with medication adherence and management, especially for those requiring complex instructions such as prednisone tapers. Another clinician requested a mechanism for annotating medications on the dashboard, for example, to explain why it was discontinued.

#### 3.2.4 Concerns about the feasibility of building and using the dashboard

Perhaps the most serious concerns expressed by clinicians were around the availability and accuracy of the EHR data to be imported into the dashboard (Table 3E). All of the clinicians worried about the accuracy of the medication list in the EHR and the availability of laboratories, especially those performed outside of the institution. Most clinicians were also concerned about how using the dashboard would fit into their existing workflows and affect their time management (Table 3F). They worried it would raise a large number of questions from patients that would lengthen the visit. Some mentioned that having medical assistants review the dashboard with patients while checking vital signs before the clinical visit began physicians might be a way to save time.

## 4 DISCUSSION

In this qualitative study of a diverse group of RA patients, including English and Spanish speakers and patients with adequate or limited health literacy alongside clinicians, we assessed the acceptability of a series of RA dashboard prototypes. After several iterations, we found a high level of patient and clinician acceptance for an RA dashboard across all groups of patients (ie, positive reactions to using the dashboard and the absence of refusals to use it). Patients and clinicians anticipated that the final dashboard could enhance communication around RA disease activity and improve patient self‐management. Patients saw the dashboard as valuable for communicating with their rheumatology clinicians, and also for coordinating care between different medical specialists and helping their families better understand their disease. Clinicians were also enthusiastic about using a dashboard during their visits; they seemed to underestimate the extent to which patients wanted detailed information about their disease, including access to doctor's notes and interpretations. Although most clinicians believed the dashboard could enhance their ability to communicate with patients, they were sceptical that the dashboard would work as intended, and anticipated difficulties in importing accurate data from the EHR and incorporating it effectively into their workflows.

Few studies have examined the impact and feasibility of using health information technology (IT) to improve communication around RA PROs during ambulatory visits, and few RA‐specific dashboards have been developed to date.^3^, ^9^, ^10^, ^11^ These platforms aggregate similar data as our RA dashboard but were not designed to be patient‐facing. In 2010, the French Society of Rheumatology launched Sanoia, a web‐based platform specifically designed for patients to track and monitor their RA symptoms and disease activity.^22^ Although Sanoia seemed to make improvements in patient‐clinician communication, a quarter of patients never accessed the tool, and use declined after 1 year. Our RA dashboard is unique in that it was designed in collaboration with RA patients to be patient‐facing, with the purpose of improving patient‐clinician communication and enhancing patients’ understanding of their disease.

Themes from patient focus groups illustrated the strength of an iterative, human‐centred design process. Although we engaged the intended end users at every stage of development, we continued to gain new insights around design preferences for data presentation during focus groups. The dashboard was initially designed to only present the most essential data using simple language and iconography, based on previous patient feedback.^13^ Yet many patients expressed difficulty interpreting the dashboard, irrespective of their health literacy level. At the same time, patients from both adequate and limited literacy groups objected to the ‘childish’ representation of the data and were interested in increasing the amount of detail presented. These divergent views on dashboard design highlight the need for customizable elements when developing a tool for RA patients. While initial iterations of the RA dashboard primarily featured numeric graphs that depicted trends in disease activity, pain and functional status, some patients requested more visual depictions, including representations of the body. Designers then incorporated a homunculus as a pictorial representation of disease activity, which many subsequent focus group participants identified as their preferred prototype. Importantly, these preferences could not be reliably predicted by level of health literacy or language group. This may be explained by differences in numeracy or graphical literacy, which we did not formally assess. A previous study on the types of literacy skills needed to comprehend a medical dashboard showed that there is variability in numeracy and graphical literacy even among patients with adequate health literacy.^23^

Limited health literacy and monolingual Spanish‐speaking patients are particularly vulnerable to suboptimal patient‐clinician communication. Previous studies have shown that RA patients with limited health literacy and limited English proficiency are more likely to report suboptimal shared decision‐making when interacting with their clinicians^24^ and that these patients are particularly vulnerable to adverse outcomes,^25^, ^26^, ^27^ highlighting the importance in facilitating patient‐clinician communication in this particular population. Engaging a diverse group of patients, as we did here, revealed important connotative meanings that would not have otherwise been considered during the design process. For example, one patient recommended inverting the CDAI scale because the ‘good’ value should be represented on the top. Patient focus groups also generated innovative ideas for expanded capabilities in a dashboard, such as a journaling function to help track self‐management strategies. End‐user participation is critical in designing a tool that will have a high likelihood of being adopted by its intended audience.^28^

This study has several strengths. First, the iterative, human centred‐design process engaged end‐users at every stage of development. We included a diverse group of patients, including Spanish‐speaking patients. Native Spanish speakers facilitated and analysed data from the Spanish‐language groups. We included adequate and limited health literacy patients in the focus groups, which builds confidence in our findings of utility and acceptability of the tool. Facilitators specifically designed the focus groups to solicit patient and clinician concerns about the tool or its use in clinic. As a result, we are more confident that we have a realistic view of stakeholder perspectives on the implementation of the dashboard.

There are some limitations to this study as well. First, as is the case for all small studies, the generalizability of these findings is unknown. We included clinicians and diverse patients from multiple health systems to improve the potential applicability of these findings to other settings, but more research will be needed to confirm our findings outside of this institution. Second, we cannot be sure from the focus group data whether the RA dashboard will, once implemented, significantly change patients’ health behaviours, such as improving medication adherence, exercise or other self‐management strategies, although some patients predicted it might. Our hypothesis is that by improving patient‐clinician communication around RA disease activity, use of the dashboard will promote shared decision‐making, which would ultimately improve patient knowledge and engagement, and perhaps medication adherence and patient outcomes.^29^ Further studies are needed to evaluate patient activation once the RA dashboard has been piloted.

Work is currently underway to implement the RA dashboard into our local EHR. The dashboard will be launched from inside the patient's chart in the EHR during the face‐to‐face visit with the clinician, and data elements will be automatically populated from real‐time EHR data. We plan for the dashboard to be curated—that is, it will be displayed on the screen in the examination room and its elements can be explained by the clinician; it can serve as a jumping‐off point for a conversation between the patient and the clinician in order to support shared decision‐making.

Informed by the findings of this study, we have designed the RA dashboard to be partially customizable to patient and clinician preferences. Specifically, for patients, we have made each section of the dashboard expandable and collapsible so that only one section can be ‘expanded’ at a given time. Additional ideas for future iterations of the dashboard include making it possible for patients to contribute additional information or annotations to the visualizations, such as information about exercise, mood, or sleep; to change the time range of the graphs to the most relevant period for an individual patient; or to edit the ‘goal’ green zone for each PRO based on their personalized targets. Explanatory ‘pop‐ups’ in each section of the dashboard could also be tailored to the patient's preferences. Training videos or tutorials may need to be developed in order to enhance patient understanding of the RA dashboard. We also anticipate that the RA dashboard could be made accessible to patients through the online patient portal. On the clinician's side, we can make it possible to add other ‘widgets’ to the dashboard, including other useful information such as X‐ray results, osteoporosis screening results, or relevant vaccination information. Future studies should focus on how different types of patients or clinicians engage with the dashboard and on evaluating the impact the RA dashboard will have on patient care, particularly on disease outcomes, medication adherence and patient self‐management.

In sum, using principles of human‐centred design, we created a RA dashboard that is well‐accepted among RA patients, although clinicians remained sceptical about its implementation. The ability to customize data display preferences is important in tailoring the dashboard to patients with diverse needs and preferences. Special attention should be given to feasibility concerns voiced by clinicians.

## CONFLICTS OF INTERESTS

The authors report no conflict of interests.

## Data Availability

The data that support the findings of this study are available from the corresponding author upon reasonable request.
